# Evaluation of the relationship between Ki67 expression level and neoadjuvant treatment response and prognosis in breast cancer based on the Neo-Bioscore staging system

**DOI:** 10.1007/s12672-023-00809-w

**Published:** 2023-10-24

**Authors:** Yurdanur Sullu, Leman Tomak, Guzin Demirag, Bekir Kuru, Necati Ozen, Filiz Karagoz

**Affiliations:** 1 Department of Pathology, Faculty of Medicine, Ondokuz Mayis University, 55139 Samsun, Turkey; 2 Department of Biostatistics and Informatics, Faculty of Medicine, Ondokuz Mayis University, Samsun, Turkey; 3 Department of Medical Oncology, Faculty of Medicine, Ondokuz Mayis University, Samsun, Turkey; 4 Department of Surgery, Faculty of Medicine, Ondokuz Mayis University, Samsun, Turkey; 5 Department of Surgery, Medical Park Hospital, Samsun, Turkey

**Keywords:** Breast carcinoma, Ki67, Neo-Bioscore, Neoadjuvant chemotherapy, Pathologic response

## Abstract

**Background:**

Neoadjuvant chemotherapy (NAC) is widely used in the treatment of primary breast cancer. Different staging systems have been developed to evaluate the residual tumor after NAC and classify patients into different prognostic groups. Ki67, a proliferation marker, has been shown to be useful in predicting treatment response and prognosis. We aimed to investigate the prognostic importance Neo-Bioscore stage and pretreatment and posttreatment Ki67 levels in breast cancer patients who received NAC and correlations between Neo-Bioscore stage and pretreatment and posttreatment Ki67 levels.

**Methods:**

A total of 176 invasive breast carcinoma patients who underwent NAC were included in the study. Ki67 levels were evaluated by immunohistochemical methods in Trucut biopsy and surgical excision specimens. Patients were classified into prognostic groups using the Neo-Bioscore staging system.

**Results:**

Patients with high pretreatment Ki67 score were more likely to be in the higher Neo-Bioscore risk group (p < 0.001). Patients with a high posttreatment Ki67 score were more likely to be in the higher Neo-Bioscore prognostic risk group (p < 0.001). Overall survival (OS) and disease-free survival (DFS) were shorter in patients with high posttreatment Ki67 scores and in patients in the higher Neo-Bioscore risk group. We also determined a cutoff 37% for pathological complete response.

**Conclusion:**

Neo-Bioscore staging system is found to be important in predicting survival. The posttreatment Ki67 level is more important than pretreatment Ki67 level in predicting survival.

## Introduction

Breast cancer is the most common cancer in women worldwide [1]. Neoadjuvant chemotherapy (NAC) is applied as a standard treatment to reduce the tumour size and perform a more limited surgery in high-risk operable patients as well as inoperable patients [2]. NAC response is a prognostic determinant in the long term, and it is also useful in assessing the effectiveness of treatment in the short term. It allows the detection of tumors unresponsive to treatment at an early stage and the discontinuation of ineffective treatment and/or the addition of other treatments [3]. While pathological complete response (pCR) is the end point used in the assessment of treatment efficacy and is generally reported to be correlated with favorable survival, it is not associated with good prognosis in all breast cancers [4, 5]. Assessment of residual disease after NAC is important to identify patients who should receive additional adjuvant therapy, as well as to identify the subgroup with good prognosis among patients with this residual disease [5]. For this purpose, prognostic scoring systems have been developed to separate breast cancer patients into different prognostic groups after NAC [6–8]. There are many grading systems that evaluate the pathological response, and these systems evaluate only the breast or the breast and axilla together [6–8]. The prognostic importance of pCR remains controversial due to differences in evaluation methods and the prognoses of subgroups [4]. The correlations of biological characteristics of the tumor with treatment response and course of disease have been demonstrated in many studies [9].

The Neo-Bioscore is a prognostic model developed to assess the risk of recurrence in women with breast cancer undergoing NAC. The Neo-Bioscore is a staging system that considers clinical stage at the time of diagnosis, pathological stage after NAC, estrogen receptor (ER) status, grade and HER2 status. The Neo-Bioscore is calculated as the sum of the tumor grade, ER, HER2 and clinical and pathological stage scores [9]. The Neo-Bioscore includes both pretreatment and posttreatment disease burden and tumor biological factors [9, 10].

As in all cancers, proliferation is an important characteristic of breast tumors [11, 12]. Ki67 is a well-known proliferation marker used to assess cell proliferation. Ki67 assessment is most commonly performed by detecting the Ki67 antigen, which is expressed in all phases of the cell cycle except G0, by an immunohistochemical method with an anti-Ki67 monoclonal antibody [12, 13]. The prognostic and predictive value of Ki67 has been investigated in many studies. Although it has been criticized for low reproducibility, many studies have shown that Ki67 is a prognostic and predictive marker [11, 12, 14–16]. Ki67 is a useful clinical marker for subtype classification, prognostication, and predicting therapeutic response in breast cancer [17, 18]. The effect of the Ki67 level on NAC is complex [11, 19, 20]. It has been reported that pretreatment Ki67 levels correlate with treatment response, and tumors with higher Ki67 levels respond better to treatment. There is no definite threshold value that will determine which patients will achieve pCR and which will have pathological nonresponse (pNR) to treatment [20–22]. Although many studies have shown that the pretreatment Ki67 level is prognostic, some studies have reported that evaluating Ki67 after NAC can provide more accurate prognostic information [19, 20, 23].

In this study, we aimed to investigate the prognostic importance Neo-Bioscore stage and pretreatment and posttreatment Ki67 levels in breast cancer patients who recived NAC and correlations between Neo-Bioscore stage and pretreatment and posttreatment Ki67 levels.

## Materials and methods

One hundred seventy-six patients who underwent NAC with a diagnosis of invasive breast carcinoma at Ondokuz Mayıs University Faculty of Medicine Hospital between 2013 and 2020 and who had a Trucut biopsy pretreatment and a posttreatment surgical specimen were included in the study. The study was approved by Clinical Research Ethics Committee of Ondokuz Mayis University that waived the informed consent of this study (11.03.21/B.30.2.ODM.0.20.08/144-399). NAC was applied according to the protocols discussed on a case-by-case basis by the multidisciplinary tumor council. NAC was administered to patients with tumors larger than 2 cm or axillary lymph node positivity or triple-negative breast cancer (TNBC). Especially in hormone receptor-positive HER2-negative patients, it was preferred that the size be above 2 cm. Generally, regimens consisting of 4 cycles of doxorubicin, cyclophosphamide (dose-intensive) and weekly paclitaxel (12 cycles) were applied. Trastuzumab was added to the treatment after 4 cycles of doxorubicin and cyclophosphamide in HER2-positive patients. All patients underwent physical examination, mammogram and ultrasound. Coils were placed in all patients before NAC.

### Pathological evaluation

Pretreatment biopsies and posttreatment surgical specimens were reviewed. Biopsy samples were immunostained and evaluated for the expression of ER, progesterone receptor (PgR), HER2 and Ki67. pCR was defined as the absence of invasive tumor breast and axilla.

The Neo-Bioscore was calculated by scoring clinical stage and pathological stage from 0 to 2, and cases with ER negativity, grade 3 disease and HER2 negativity were given an additional 1 point. Clinical stages I and IIA and pathological stages 0 and I were scored as 0, clinical stages IIB and IIIA and pathological stages IIA, IIB, IIIA, and IIIB were scored as 1, and clinical stage IIIC and pathological stage IIIC were scored as 2 [9]. Neo-Bioscores, reflecting scores from 8 categories and ranging from 0 to 7, were classified into 4 cluster Neo-Bioscore groups: scores between 0 and 3 were classified as low, scores between 4 and 5 were classified as low-intermediate, a score of 6 was classified as high-intermediate, and a score of 7 was classified as high-risk as suggested by Resende et al. [24].

ER, PgR, HER2 and Ki67 immunostaining was performed and evaluated in patients with residual tumors. The slides, which were evaluated by the breast pathologists involved in the study, were reviewed. The histological type, histological grade, ER, PgR, and HER2 status and Ki67 level of the tumor tissue in biopsy samples were recorded. Tumors were divided into molecular subgroups as luminal A, luminal B HER2-negative, luminal B HER2-positive, HER2-positive and triple negative according to the ER, PgR, HER2 and Ki67 results [17]. The histological type of the tumor, histological grade, tumor size, lymph node status, Ki67 level in surgical specimens containing the residual tumor, Neo-Bioscore and clustered Neo-Bioscore of all surgical specimens were recorded. The clinical stage information from the initial appointment and overall survival (OS) and disease-free survival (DFS) data were obtained from the records of the medical oncology department. DFS was defined as the date of the first histological diagnosis to the date of the first recurrence of breast cancer at any site. OS was defined as the date of the first histological diagnosis to the date of death from any cause.

### Immunohistochemical study

All immunohistochemical studies were performed with an automatic immunostaining device (Ventana Benchmark XT, Ventana Medical Systems, France and Ventana Benchmark Ultra, Ventana Medical Systems, Tucson, Az, USA) according to the company's protocol. The primary antibodies used were anti-ER rabbit monoclonal primary antibody (clone SP1, Ventana), anti-PgR rabbit monoclonal primary antibody (clone 1E2, Ventana), anti-HER2/neu rabbit monoclonal antibody (clone 4B5, Ventana), and anti-Ki67 rabbit monoclonal primary antibody (clone 30-9, Ventana). All antibodies were ready to use. One percent nuclear staining was considered the cutoff for ER and PgR positivity, and staining less than 1% was considered negative [25]. For ER, staining between 1 and 10% was scored as low positive, and staining between 11 and 100% was scored as positive according to the ASCO/CAP guidelines [25]. However, since there were only 4 patients in the low-positive group, the low-positive and positive patients were grouped as positive. HER2 expression evaluation was performed according to the ASCO/CAP 2018 guidelines, and HER2 positivity (score 3) was immunohistochemically defined as complete, intense membrane staining in more than 10% of tumor cells [26]. Silver in situ hybridization (SISH) was performed on an automated stainer (Ventana Benchmark XT, Ventana Medical Systems, France and Ventana Benchmark Ultra, Ventana Medical Systems, Tucson, Az, USA) using a dual SISH probe (INFORM HER2 Dual ISH DNA Probe Cocktail, Ventana) on immunohistochemistry 2 + samples. HER2 status was evaluated according to the 2018 ASCO/CAP guidelines [26]. Ki67 expression was evaluated by counting 500–1000 cells, depending on tumor cellularity, in at least 3 high-power fields (× 40), provided that at least 500 cells were counted. Hot spot areas in tumors with heterogeneous staining were also evaluated and scored. Ki67 expression was calculated as the ratio of stained cells to total tumor cells [13]. Cases with 5% or less staining were scored as 1, cases with 6–29% staining were scored as 2, and cases with 30% or more staining were scored as 3 [27]. In cases where a small number of residual tumor cells (less than 500) remained after treatment, all tumor cells were counted and scored.

### Statistical methods

Statistical analyses were performed with SPSS 21.0 for Windows. Data are presented as the mean ± standard deviation (SD) or as the median (min–max) as a frequency (%). The Shapiro–Wilk test was used to analyze the quantitative outcomes assumed to have a normal distribution. Nonnormally distributed data were analyzed by the Mann–Whitney test. The relationship between variables was assessed by Spearman rank correlation for nonnormally distributed data. The area under the ROC curve (AUC) was used as to evaluate a diagnostic test's discriminatory power. Confidence intervals were computed for AUC analysis. In this article, sensitivity and specificity were evaluated. The Kaplan‒Meier method with the log-rank test was used to identify significant differences for survival analysis. A p value less than 0.05 was considered statistically significant.

## Results

All 176 patients included in the study were women. The mean age of the patients was 51.0 $\pm$ 11.5 years (23–81 years). The mean follow-up period was 46.1 $\pm$ 18.5 months (12–116 months). The clinicopathological characteristics of all patients are summarized in Table 1.

**Table 1:** Patient and clinicopathological characteristics (n = 176)

Characteristic	n = 176 (%)
Age (year, mean, range)	51.0 ± 11.5 (23–81)
Histology	
IDC	161 (91.5)
ILC	10 (5.7)
MIBC (IDC and ILC)	2 (1.1)
Musinous ca	1 (0.6)
Metaplastic ca	2 (1.1)
Surgery	
Breast conservative	101 (57.4)
Mastectomy	75 (42.6)
Axillary surgery	
SLN	135 (76.7)
AD	32 (18.2)
SLN + AD	9 (5.1)
ER	
Positive	122 (69.3)
Negative	54 (30.7)
HER2	
Positive	63 (35.8)
Negative	113 (64.2)
Grade	
I	3 (1.7)
II	52 (29.5)
III	121 (68.8)
Stage	
I	9 (5.1)
II	121 (68.8)
III	46 (26.1)
Molecular subtype	
Luminal A	29 (16.5)
Luminal B HER2 −	54 (30.7)
Luminal B HER2 +	39 (22.2)
HER2 +	27 (15.3)
Triple −	27 (15.3)
Lymph node	
Negative	99( 56.2)
Positive	77 (43.8)
PreNAC Ki67	
≤ 5%	7 (4.0)
6–29%	52 (29.5)
≥ 30%	117 (66.5)
PostNAC Ki67 (n = 139)	
≤ 5%	52 (37.4)
6–29%	36 (25.9)
≥ 30%	51 (36.7)

AD: Axillary dissection; ER: Estrogen receptor; IDC: Invasive ductal carcinoma; ILC: Invasive lobular carcinoma; MIBC: Mixt invasive breast carcinoma; ACC: Neoadjuvant chemotherapy; SLN: Sentinel lymph node

**Fig. 1:**
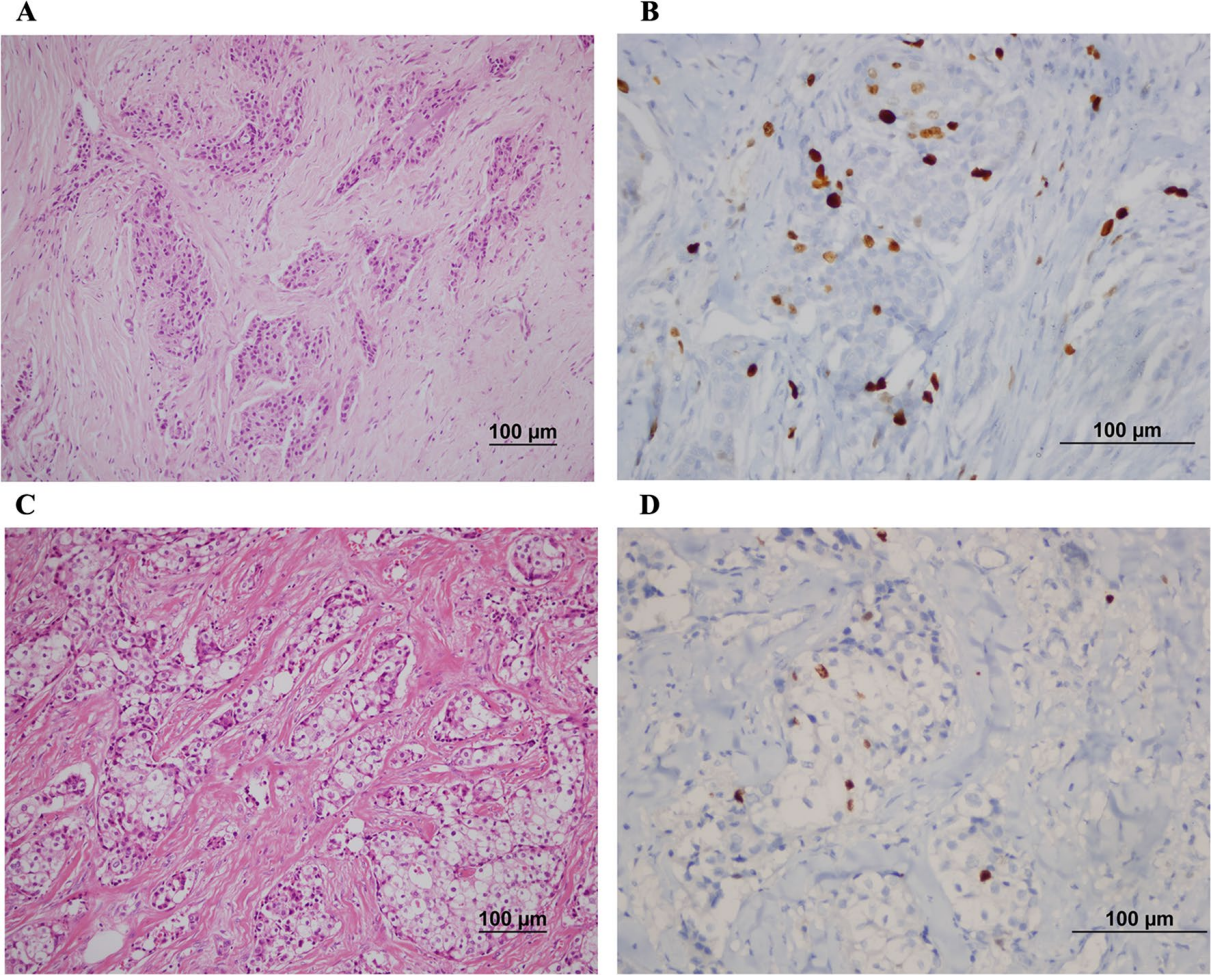
Luminal A invasive ductal carcinoma: pretreatment biopsy H-Ex200 (**A**); Ki67 X400 (**B**) (the Ki67 level was evaluated as %10); residual disease after neoadjuvant chemotherapy: H-E × 200 (**C**) and Ki67 × 400 (**D**) (the Ki67 level was evaluated as %5)

**Fig. 2:**
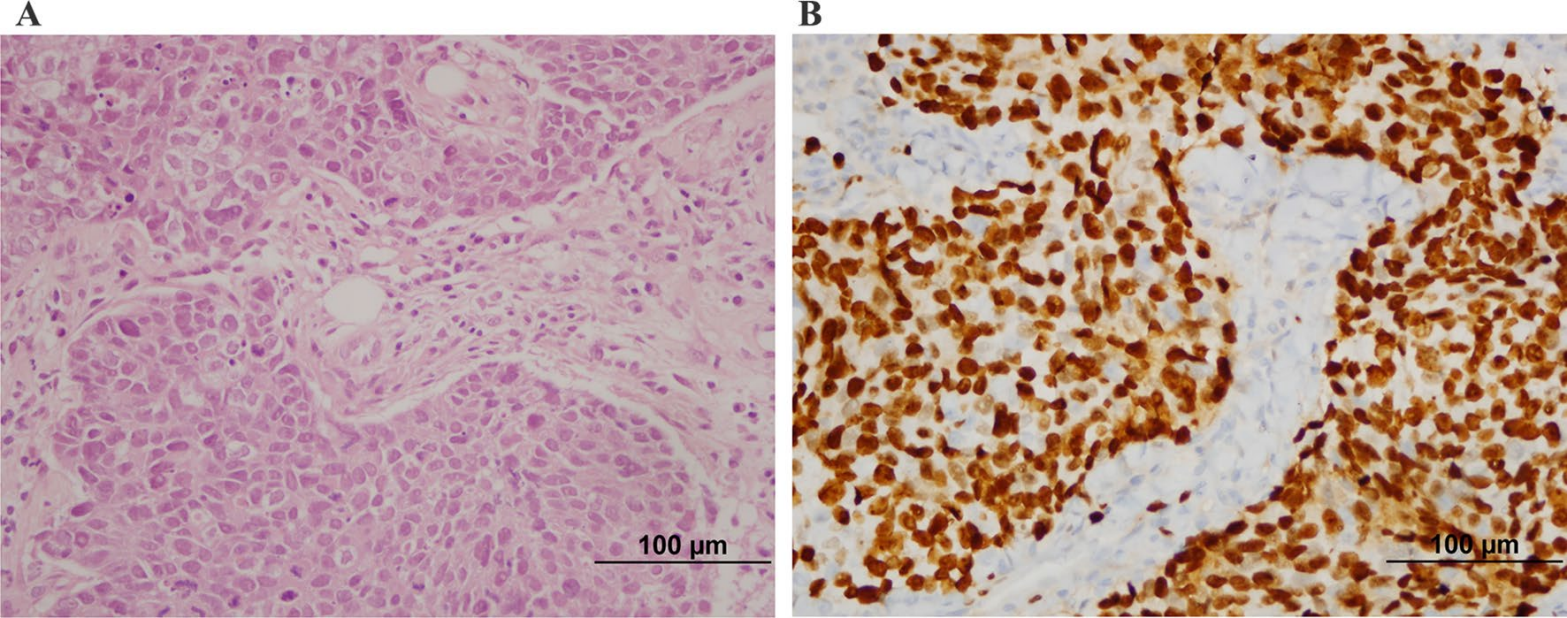
Pathological complete response of triple-negative invasive ductal carcinoma: pretreatment biopsy: H-E × 400 (**A**)and Ki67 × 400 (**B**) (the Ki67 level was evaluated as 90%)

Thirty seven out of 176 patients (21.0%) achieved pCR, no cases of complete response were observed in the luminal A subtype (Fig. 1). The highest pCR rate was observed in triple-negative tumors (51.8%), and the HER2-positive subtype had the second highest pCR rate (33.3%) (Fig. 2).

A positive correlation was found between the pretreatment Ki67 score and Neo-Bioscore risk group (r = 0.299, p < 0.001).

**Fig. 3:**
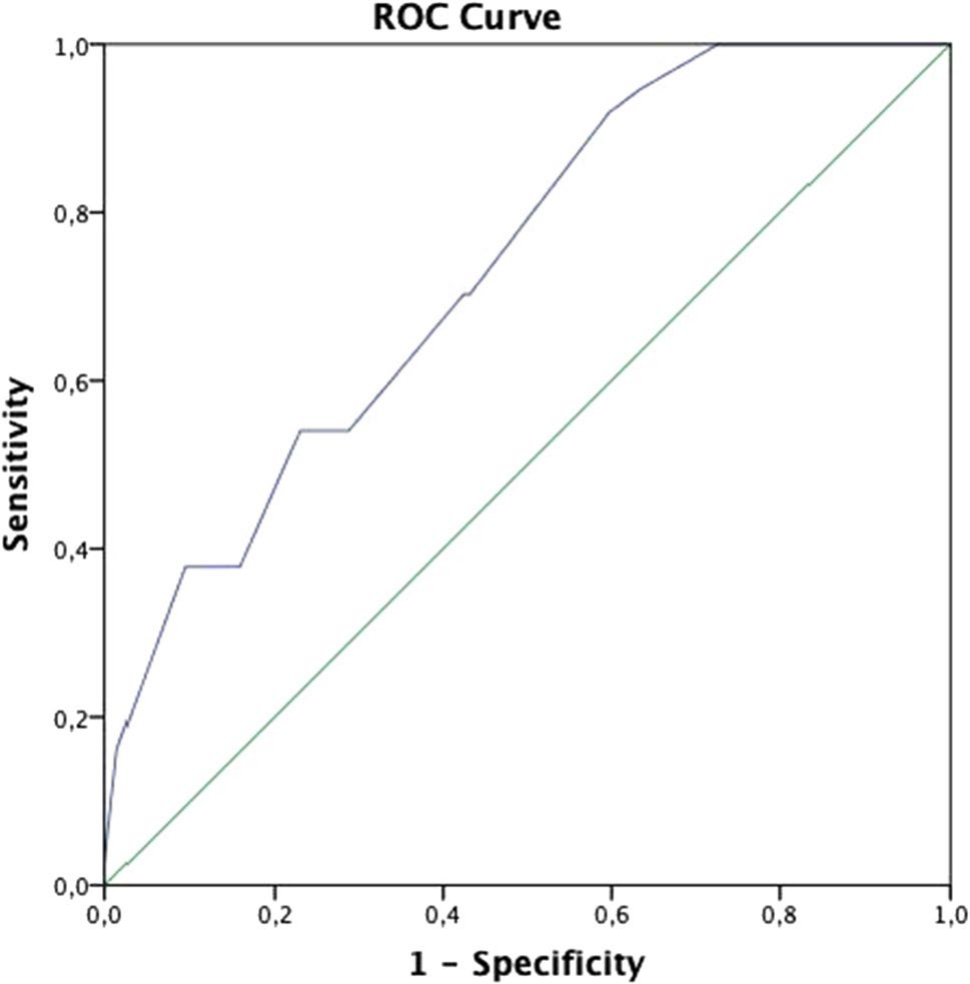
ROC curve of Ki-67 cut-off value for pathologic complete response

**Fig. 4:**
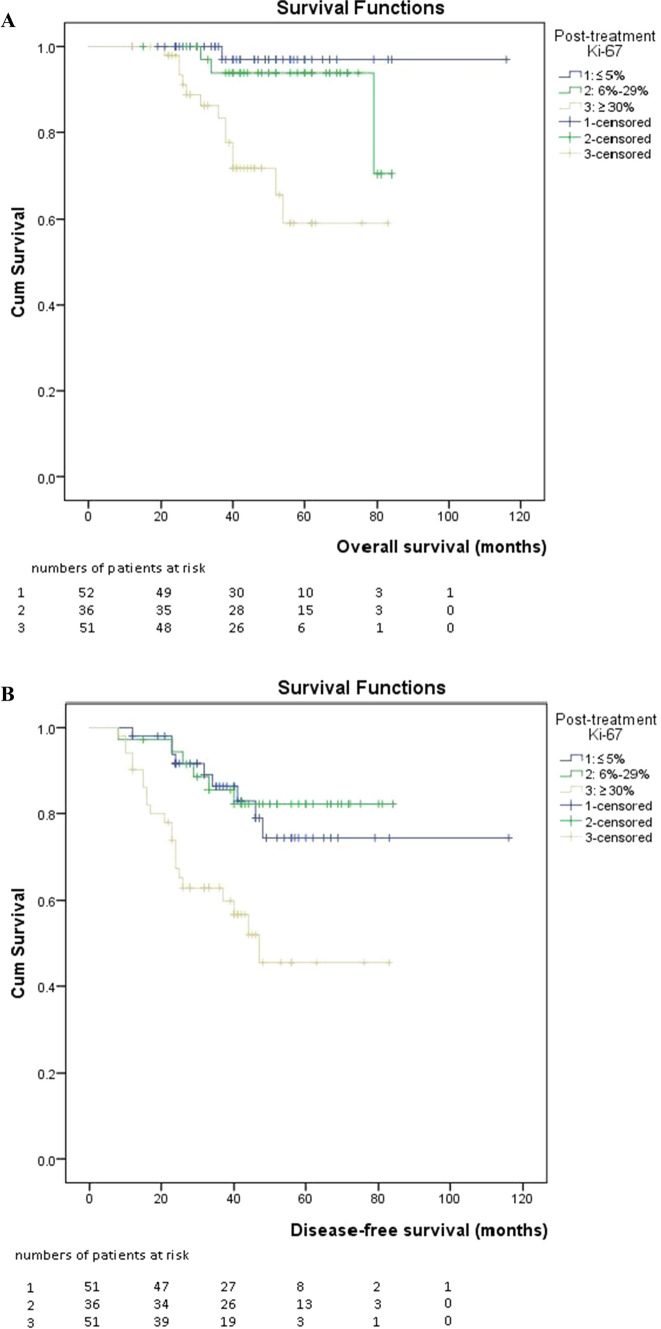
Kaplan‒Meier curve evaluating posttreatment Ki-67 level: **A** overall survival and **B** disease-free survival

When the cutoff value was taken as 37.5 for Ki67, the AUC was 0.734 in complete response group (95% CI 0.649–0.818). The value obtained was significant (p < 0.001). The sensitivity was 70%, and the specificity was 58% (Fig. 3). A positive correlation was found between the posttreatment Ki67 score and Neo-Bioscore risk group (r = 0.371, p < 0.001). A statistically significant difference was found between the posttreatment Ki67 score groups in terms of OS and DFS (p < 0.001 and p < 0.001) (Fig. 4). OS and DFS were 114 months (95% CI 109–118) and 95 months (95% CI 88–107), respectively, in the score 1 group (Ki67 ≤ %5), 80 months (95% CI 75–84) and 74 months (95% CI 66–81) in the score 2 group (Ki67 6–29%), and 65 months (95% CI 57–73) and 52 months (95% CI 42–62) in the score 3 group (Ki67 ≥ 30%). No statistically significant difference was found between the pretreatment Ki67 score groups in terms of OS and DFS (p** > **0.05). When evaluated patients grouped by intrinsic subtype, negative correlations were found between the pretreatment Ki67 score and OS and DFS (r = − 0.331, p = 0.014; r = − 323, p = 0.017, respectively) and a negative correlation between posttreatment Ki67 and DFS were found in the luminal B HER2-negative subtype (r = − 0.298, p = 0.040).

There was a statistically significant difference in OS and DFS between the Neo-Bioscore risk groups (p = 0.008, p < 0.001). DFS was 81.4 months (95% CI 75.3–87.6) in the low-risk group, 79.5 months (95% CI 67.3–91.8) in the low-intermediate-risk group, 59.0 months (95% CI 40.3–77.6) in the high-intermediate-risk group, and 10 months (95% CI 10–10) in the high-risk group.

In the OS evaluation, the survival times in the low- and low-intermediate-risk groups were similar, being a mean of 91.6 (95% CI 86.3–96.8) and 92.8 (95% CI 82.0–103.6) months, while OS was 59.3 (95% CI 41.0–77.5) months in the high-intermediate group. There was only one patient in the high-risk group, and the survival time was 10 months.

## Discussion

Since NAC is currently an accepted treatment for breast cancer, including early-stage breast cancer, markers with prognostic and predictive value for NAC have been studied [21, 23]. In this study, we demonstrated that Neo-Bioscore staging and posttreatment Ki67 score can provide prognostic infotmation about OS and DFS. In addition, we found a correlation between the pretreatment and posttreatment Ki67 scores and Neo-Bioscore risk groups. As many studies have shown improved long-term outcomes in patients who achive pCR after NAC, many staging sytems have been proposed including assessment of treatment response [4, 28, 29]. In some studies, only the response of the breast is taken into account, while in others, the lymph node response is also taken into account along with the breast response [6, 7, 30]. However, there is no agreement on which staging system is optimal to use after NAC [10]. The relationships between biological characteristics of the tumor and treatment response and disease prognosis have been demonstrated [23, 31]. Therofore, response scoring systems have been developed that take into account the biological characteristics of the primary tumor as well as the disease burden when predicting prognosis after NAC [7, 9, 10, 32]. The prognostic significance of pCR varies considerably according to breast cancer subtype [4, 33, 34]. It has been shown that scoring system created by adding grade and ER status to pretretment clinical stage and posttreatment pathological stage (CPS + EG) are more successful in predicting survival [8, 32, 35]. However, the scoring system is not useful for determining the prognosis of HER2-positive tumors, because it does not take into account the response of HER2-positive tumors to trastuzumab plus NAC [32]. The Neo-Bioscore includes the addition of HER2 status to grade, estrogen status and clinicopathological stage [9]. Neo-Bioscore is more successful in categorizing patients into prognostic subgroups [9, 35, 36]. Bergquist et al., in their study consisting of 12,002 patients, reported that CPS + EG and the Neo-Bioscore were superior to AJCC clinical and pathological stage in determining prognosis, and the Neo-Bioscore had the highest prognostic value [36].

Luminal A-like tumors are low-proliferation tumors and have a good prognosis, although the pCR rate is low [37]. It is known that pCR is not a good prognostic marker in these tumors, whereas it is a good prognostic marker in aggressive tumors such as HER2-positive and triple-negative tumors [4, 37]. The Neo-Bioscore can be used to determine the prognosis of the patients of all disease subtypes that have received NAC. Thus, it is more successful in assessing globally without categorizing tumors into subgroups specifically [8]. It has been suggested that the Neo-Bioscore grading system could not successfully determine the prognosis of HER2-positive patients who were not treated with trastuzumab [38]. Therefore, a modified Neo-Biocsore stage system has been proposed [38]. All of our HER2-positive patients received trastuzumab.

The importance of the Ki67 expression level as a prognostic factor in breast cancer has been demonstrated in many studies [18, 20, 23]. It has been reported that tumors with a high pretreatment Ki67 index have a higher pCR rate [20]. Balmativola et al. found that the cutoff for the Ki67 index to distinguish pNR patients from pPR and pCR patients was 18% [21]. We identified a Ki67 cutoff for pCR of 37%. We also determined that the Neo-Bioscore of patients with a high pretreatment Ki67 score was also high, and the Ki67 score was correlated with the Neo-Bioscore.

Luminal B HER2-negative tumors are tumors of an intermediate category; endocrine therapy is recommended for all patients with luminal B HER2-negative disease, while cytotoxic therapy is recommended for the majority of these patients [18]. It has been shown that patients with luminal B HER2-negative tumors with a high proliferation index benefit from chemotherapy [39]. Similar to our study, Zong et al. also reported that the DFS of luminal B HER2-negative tumors with a high Ki67 index was shorter than that of patients with a low Ki67 index [40]. However, they did not observe a correlation between OS and the Ki67 index, unlike our study [40]. Ki67 should be considered for the selection of more aggressive treatment modalities in luminal B HER2-negative tumors [40].

The evaluation of residual disease after NAC is important not only for identifying patients who should receive additional adjuvant therapy but also for identifying the subgroup with a good prognosis among patients with residual disease [5, 13]. It has been reported that posttreatment Ki67 expression levels are associated with prognosis [5, 13, 23]. Especially in tumors in which the prognostic value of pCR is limited, a importance of a prognostic predictor such as Ki67 that can be evaluated in residual tumors is being increasingly recognized. It has been reported that patients with low Ki67 levels in residual tumors after NAC have a similar prognosis to patients with pCR [41]. In our study, the OS and DFS of the patients with a low posttreatment Ki67 score were longer than those of patients with a high posttreatment Ki67 level. In the intrinsic subtype analysis, we found a correlation between posttreatment Ki67 score and DFS in the luminal B HER2-negative subtype. It has been reported that the residual proliferative cancer burden (RPCB), a metric created by incorporating Ki67 to the RCB system, provided better prognostic information than Ki67 or the RCB score alone [31]. In this study, we found that the Neo-Bioscore increased as the posttreatment Ki67 score increased. These results show that a high Ki67 proliferation index in the residual tumor indicates the persistence of resistant and actively proliferating cancer cells and can be used as an important prognostic marker [9, 31].

Our study has some limitations. First, although the number of patients was not low, the number of patients in some groups based on intrinsic subtype, Neo-Bioscore risk group was limited. It is well known that tumor subtype is one of the most important prognostic factors in breast cancer [37]. Therefore, the relatively small sample size to evaluate the relationship between Ki67 score and survival in subtypes limits its statistical power. Second, longer follow-up periods are needed to evaluate the prognosis of intrinsic subtypes, especially for those for which long-term prognosis is important.

In conclusion, this study showed that the posttreatment Ki67 level is more important than pretreatment Ki67 level in predicting survival. Among the intrinsic subtypes of breast cancer, the luminal B HER2-negative subtype with high pretreatment and posttreatment Ki67 levels had a poor prognosis. The Neo-Bioscore staging is found to be important in predicting survival. Classification systems that include evaluation of biological characteristics of the tumor, including Ki67 levels, will provide more accurate prognostic information.

## Data Availability

The datasets generated and analysed during the current study are available from the corresponding author on reasonable request.
